# Scalable Production of Glioblastoma Tumor-initiating Cells in 3 Dimension Thermoreversible Hydrogels

**DOI:** 10.1038/srep31915

**Published:** 2016-08-23

**Authors:** Qiang Li, Haishuang Lin, Ou Wang, Xuefeng Qiu, Srivatsan Kidambi, Loic P. Deleyrolle, Brent A. Reynolds, Yuguo Lei

**Affiliations:** 1 Department of Chemical and Biomolecular Engineering, University of Nebraska, Lincoln, Nebraska, USA; 2 Department of Bioengineering, University of California, Los Angeles, Los Angeles, California, USA; 3 Mary and Dick Holland Regenerative Medicine Program, University of Nebraska Medical Center, Omaha, Nebraska, USA; 4 Fred & Pamela Buffett Cancer Center, University of Nebraska Medical Center, Omaha, Nebraska, USA; 5 Nebraska Center for Materials and Nanoscience, University of Nebraska, Lincoln, Nebraska, USA; 6 Nebraska Center for the Prevention of Obesity Diseases, University of Nebraska, Lincoln, Nebraska, USA; 7 Department of Neurosurgery, University of Florida College of Medicine, McKnight Brain Institute, Gainesville, Florida, USA

## Abstract

There is growing interest in developing drugs that specifically target glioblastoma tumor-initiating cells (TICs). Current cell culture methods, however, cannot cost-effectively produce the large numbers of glioblastoma TICs required for drug discovery and development. In this paper we report a new method that encapsulates patient-derived primary glioblastoma TICs and grows them in 3 dimension thermoreversible hydrogels. Our method allows long-term culture (~50 days, 10 passages tested, accumulative ~>10^10^-fold expansion) with both high growth rate (~20-fold expansion/7 days) and high volumetric yield (~2.0 × 10^7^ cells/ml) without the loss of stemness. The scalable method can be used to produce sufficient, affordable glioblastoma TICs for drug discovery.

The most recent cancer cell theory attributes the aggressive phenotype, drug resistance, and recurrence of glioblastoma, the most common and aggressive brain tumor type, to the existence of tumor-initiating cells (TICs), a small population of tumor cells within the tumor mass that have stem cell properties^1^,^2^,^3^. Through a variety of mechanisms, glioblastoma TICs can survive the current radiation and chemotherapy regime, proliferate and differentiate to initiate new tumors^4^. New therapies that can eliminate glioblastoma TICs, such as killing or differentiating TICs, or sensitizing TICs to current treatment regimes appear to offer hope to treat and potentially cure glioblastoma^4^.

Primary glioblastoma TICs have been successfully isolated and cultured for long term, maintaining their capability for self-renewing^1^,^5^,^6^,^7^,^8^,^9^,^10^. Similar to normal neural stem cells (NSCs), cultured glioblastoma TICs can be differentiated into astrocytes, neurons and oligodendrocytes. Following xenotransplantation, glioblastoma TICs can form tumors with structures similar to the primary tumors. These cultured glioblastoma TICs are invaluable for developing new drugs that can induce their death or differentiation, or sensitivity to current therapies. Drug discoveries require very large numbers of cultured cells^11^,^12^,^13^,^14^. For instance, about 1 × 10^10^ TICs are needed to screen a one-million-compound library one time with the 384-well plates. And recent advances in combinatorial chemistry and noncoding RNAs have given rise to many large libraries that can be screened^15^. Cost-effective production of glioblastoma TICs in large scale, however, remains a significant challenge.

Currently, glioblastoma TICs are either cultured as 2 dimension (2D) adherent monolayer or as 3 dimension (3D) neurospheres^1^,^5^,^6^,^7^,^8^,^9^,^10^. While these methods can generate sufficient cells for basic science research, both are limited in their ability to produce large numbers of cells required for drug discovery and screening. Research has demonstrated that 2D culture systems, which suffer from inherent heterogeneity and limited scalability and reproducibility, are not suitable for large scale cell culture^16^. An attractive approach for scaling up production is to develop 3D culture technologies. However, the aforementioned neurosphere culture only supports glioblastoma TICs culture at low density, yielding merely ~1 × 10^6^ cells per milliliter of volume. Thus, a neurosphere culture method requires tens of liter volume to produce sufficient cells to screen million-compound library one time leading to the high cost for drug development.

In this paper we describe a new, scalable method to culture glioblastoma TICs in the form of spheroids at high volumetric yield (i.e. ~2 × 10^7^ cells/ml). Glioblastoma TICs were encapsulated and grown in 3D thermoreversible hydrogels. With these hydrogels, TICs could be cultured for long term without significant change of their phenotypes and expressions of the markers. Others have successfully cultured primary glioblastoma cells in the chemically crosslinked hydrogels for drug screening^17^. However, they have not demonstrated these hydrogels were suitable for long term and scalable cultures of primary glioblastoma cells. In this paper, we also systematically compared this new method with the 2D monolayer culture and the 3D neurosphere culture.

## Results

### 2D Adherent Culture

We first confirmed the literature result that glioblastoma TICs could be cultured as 2D adherent monolayer^1^,^5^. Two glioblastoma primary TICs lines, L0 and L1, were plated on laminin-coated tissue culture plates in the Neurocult^TM^ medium, following the published protocol^1^. Both L0 and L1 attached well to the plates and grew to about 60 to 80% confluency within 5 days (Fig. S1). Dead cells were hardly detected along the culture. Cells could be propagated for multiple passages (10 passages tested in our laboratory) without significant differentiation as shown by the expression of glioblastoma TICs marker, Nestin, in the majority of cells (Fig. S1b,d). Further confirming no differentiation, no or very few cells expressed the glial cell maker, GFAP (Fig. S1b,d). The results showed 2D adherent cultures were appropriate for the long-term maintenance and expansion of glioblastoma TICs.

### 3D Neurosphere Culture

We then confirmed the literature results that glioblastoma TICs could be propagated as 3D neurospheres^5^,^6^. Single TICs (L0 and L1) were suspended in Neurocult^TM^ medium at 5 × 10^4^ cells/ml in non-treated tissue culture flask. TICs clustered into small aggregates and grew into neurospheres with diameter ~100 to 250 μm on day 7 (Fig. S2). The spheres could be dissociated with 0.05% trypsin and propagated for multiple passages (10 passages tested in our laboratory). These results demonstrated that glioblastoma TICs could be maintained and expanded for long term as 3D neurospheres at low densities.

### 3D Spheroid Culture in Shaking Plates

Although a 3D culture system, neurosphere culture is limited by low density and volumetric yield (about 1 × 10^6^ cells/ml) for large-scale cell production. At high cell densities, TICs aggregated into large cell agglomerates. Due to the diffusion limits, the supply of growth factors, nutrients and oxygen to the cells located at the center of cell agglomerates became insufficient. Meanwhile, the metabolic waste accumulated at the centers of cell agglomerates. Both led to slow cell growth, severe cell death and uncontrolled differentiation. Since stirring or shaking is widely used in bioreactors (e.g. the stirred-tanks, spinner flasks) to reduce cell agglomeration and enhance mass transport and cell growth in mammalian cell cultures^16^, we tested if culturing glioblastoma TICs in suspension in shaking plates increased their growth and volumetric yield. L0 and L1 were seeded at two densities, 1 × 10^5^ cells/ml (low density) and 1 × 10^6^ cells/ml (high density) (Fig. S3), in the Neurocult^TM^ medium in non-adhesive plates placed on an orbital shaker at 75–90 rotation per minute (rpm). Single cells quickly associated to form small spherical aggregates (spheroids) in suspension cultures. At low seeding density, ~5.0, 10.0 and 20.0-fold expansion were achieved on days 3, 5 and 7 of the culture, generating ~0.5, 1.0 and 1.75 × 10^6^ cell/ml, respectively (Fig. S3e,f). At high seeding density, ~1.5, 2.0 and 2.0-fold expansion were achieved on days 3, 5 and 7 of the culture, generating ~1.5, 2.0 and 2.0 × 10^6^ cell/ml, respectively (Fig. S3e,f). Significant cell death was detected in the cultures, especially at high cell densities as shown by the dead cell staining (Fig. S3b,d). These results demonstrated that shaking plates did not significantly increase the volumetric cell yield in suspension cultures.

### Culturing Glioblastoma TICs in 3D Thermoreversible Hydrogels

**Figure 1:**
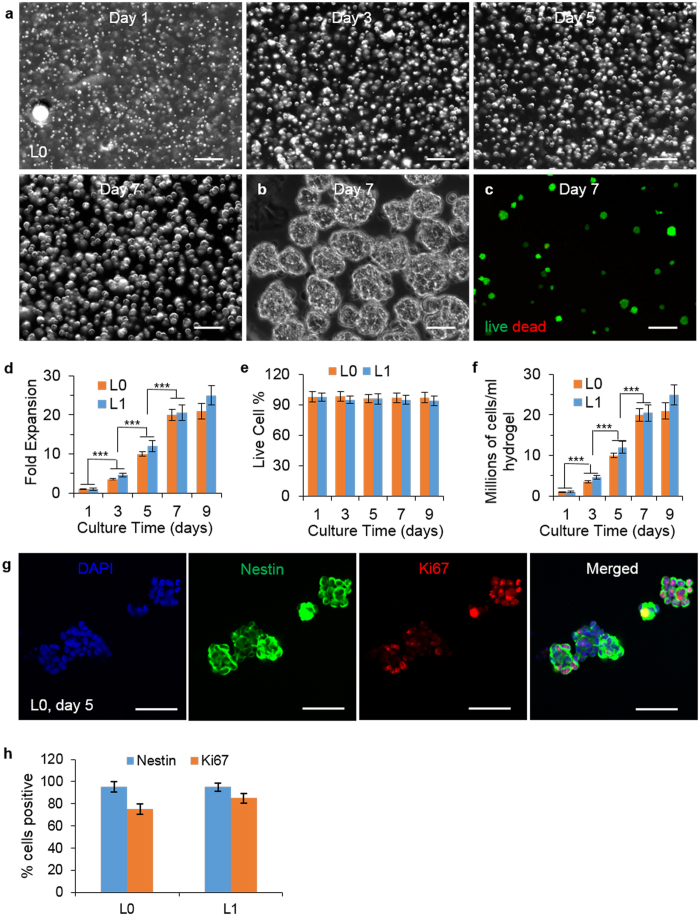
Culture glioblastoma TICs in 3D thermorevesible PNIPAAm-PEG hydrogel. (**a**) Phase images showing L0 spheroids in the hydrogel on day 1, 3, 5, 7 of the culture. (**b**) Day 7 L0 spheroids released from the hydrogel (**c**) Live (green) and dead (red) staining of day 7 L0 spheroids. The fold of expansion (**d**) cell viability (**e**) and cell density (**f**) of L0 and L1 TICs in the hydrogel on day 1, 3, 5, 7 and 9 of the culture. (**g**) Immunostaining of day 5 L0 spheroids. (**h**) Percentage of Nestin+ and Ki67+ L0 and L1 TICs after 9 days culture in the hydrogel. Images were taken after spheroids were released from the hydrogel in (**b**,**c**,**g**). Error bars represent the standard deviation (n = 3). ***Indicates statistical significance at a level of p < 0.001. Scale bar: (**a**,**c**) 250 μm; (**b**,**g**) 50 μm.

Our previous research found that cell agglomeration and shear force resulting from the medium flow were two major factors that led to the low cell growth and volumetric yield of human pluripotent stem cells (hPSCs) cultured in suspension, and that they could be eliminated by culturing hPSCs in hydrogel scaffolds^18^. Hydrogel scaffolds provide 3D spaces for cell growth and also act as physical barriers to prevent cell agglomeration and isolate the shear force. Our research showed hPSCs could be cultured at high growth rate and density in a thermoreversible PNIPAAm-PEG hydrogel long term^18^,^19^. Three features make thermoreversible hydrogels highly attractive for 3D cell culture: (A) they have rapid response to temperature change, i.e., harvesting or passaging cells can be done by simply changing temperature in the range of 4 °C to 37 °C, a mild process for cells; (B) They have good biocompatibility and low cytotoxicity; and (C) They are easily processed, i.e., into fibers or spheres through injection, modeling, extrusion or emulsion for suspension culture in large bioreactors. Thus, we explored whether glioblastoma TICs could be cultured in the PNIPAAm-PEG hydrogel. The 6–10% aqueous PNIPAAm-PEG solutions have low viscosity that allows homogenous and easy cell mixing. They form elastic hydrogels with storage modulus, G’, from 300 to 1000 Pa upon heating to 22 °C within 5 min^19^. They can be re- liquefied by cooling to 4 °C within 5 min. Preliminary studies found that glioblastoma TICs, when cultured at 1 × 10^6^ cells/ml, grew well in the 10% PNIPAAm-PEG in the Neurocult^TM^ medium. Using these tuned conditions, we achieved 10-, 20-, or 21-fold expansion and the cell viability were above 95%, with final yields of ~10.0, 20.0 and 21.0 × 10^6^ cells/ml hydrogel on days 5, 7 and 9 of the culture, respectively (Fig. 1d–f). Microscopy revealed that single seeded cells expanded and grew into spheroids (Figs 1a,b and S4a,b). Live/dead staining indicated very few dead cells within the gel or spheroids (Figs 1c and S4c). ~95% of cells were positive for the neural stem cell marker, Nestin (Figs 1g,h and S4d). The majority of cells on day 5 were proliferating as shown by the cell proliferation maker, Ki67 (Figs 1g and S4d).

**Figure 2:**
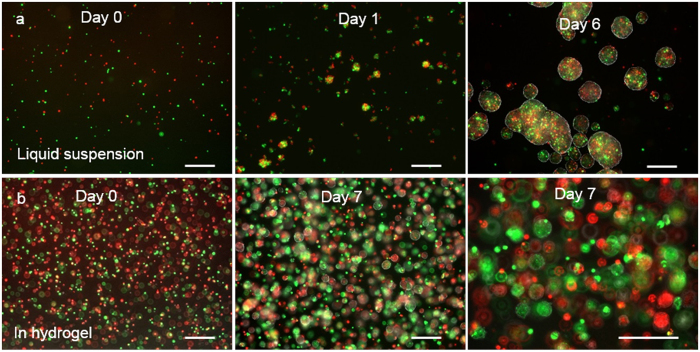
Culture glioblastoma TICs in 3D. L0 TICs stained with green or red fluorescent dye were mixed at 1:1 and cultured in suspension in liquid medium statically at 5 × 10^4^ cells/ml (**a**) or in the thermorevesible PNIPAAm-PEG hydrogel at 1 × 10^6^ cells/ml (**b**). Spheroids in the suspension culture contained both green and red cells (**a**) while spheroids in the hydrogel contained either green or red cells (**b**). Scale bar: 250 μm.

Next, we studied whether single TICs grew in the hydrogel through clonal expansion (Fig. 2). Single TICs labelled with the lipophilic tracers, DiO or DiL dye, were mixed at 1:1 and cultured either in suspension in the liquid medium (Fig. 2a) or encapsulated and cultured in the thermoreversible hydrogel (Fig. 2b) for 7 days. Spheroids in the suspension culture contained both DiO (green) and DiL (red) labeled cells, indicating the existence of spheroid fusion or merging (Fig. 2a). No or very few cell spheroids in the hydrogel contained both DiO (green) and DiL (red) labeled cells, indicating that single TICs grew through clonal expansion in the hydrogel (Fig. 2b).

**Figure 3:**
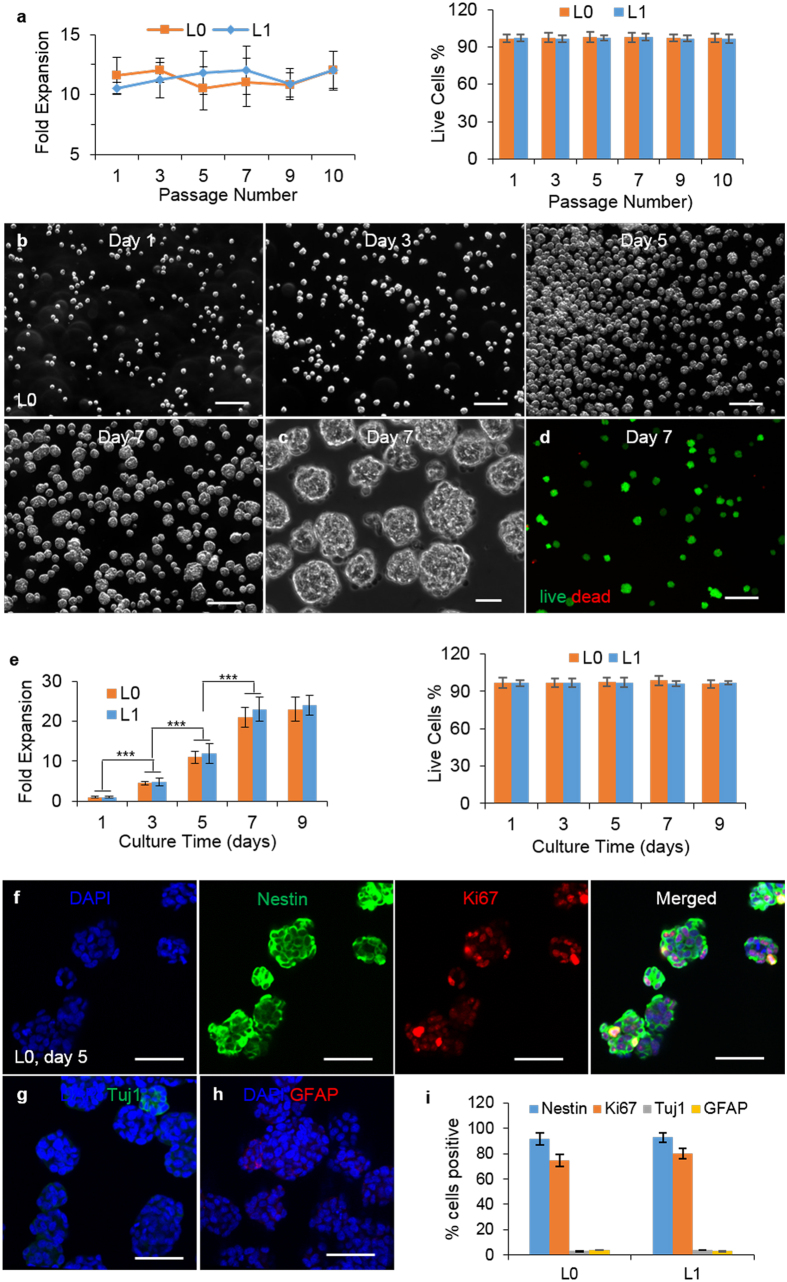
Long-term culture of glioblastoma TICs in 3D thermorevesible PNIPAAm-PEG hydrogel. (**a**) The fold expansion and cell viability per 5 days at passage 1, 3, 5, 7, 9 and 10. (**b**) Phase images showing L0 spheroids in the hydrogel on day 1, 3, 5, 7 of the culture at passage 10. (**c**) Day 7 L0 spheroids released from the hydrogel at passage 10. (**d**) Live (green) and dead (red) staining of day 7 L0 spheroids at passage 10. (**e**) The fold expansion and cell viability of L0 and L1 TICs in the hydrogel on day 1, 3, 5, 7 and 9 of the culture at passage 10. (**f**–**h**) Immunostaining of day 5 L0 spheroids and (**i**) percentage of Nestin+, Ki67+, Tuj1+ and GFAP+ L0 and L1 TICs after 9 days culture in the hydrogel at passage 10. Images were taken after the spheroids were released from the hydrogels. Error bars represent the standard deviation (n = 3). ***Indicates statistical significance at a level of p < 0.001. Scale bar: **(b**,**d**) 250 μm, (**c**,**f**–**h**) 50 μm.

We also studied whether glioblastoma TICs could be cultured long-term in hydrogel (Figs 3 and S5). Both L0 and L1 were continuously propagated for 50 days with passaging every 5 days. During each of these 10 passages, TICs expanded ~10-fold, with cell viability >95% (Fig. 3a). During this overall above 1 × 10^10^-fold expansion, >95% of cells remained Nestin+. To assess whether comparable cellular proliferation rates could be maintained over time, we re-evaluated the growth kinetics and cell viability of glioblastoma TICs after 10 passages in the gel (Fig. 3e). The expansion rate (~10-, 20-, and 21-fold at days 5, 7 and 9, respectively), neural stem cell marker expression, and spheroid size were similar to those at passage 1, indicating that the 3D hydrogel environment did not change the TICs phenotype over time (Fig. 3). To further evaluated whether this 3D hydrogel culture system has selection pressure effect for glioblastoma TICs in long-term culture, we assessed the mRNA level of the commonly used glioblastoma TICs markers CD133, CD44, CD15 and CD49f on passage 10 (P10, which was cultured in hydrogel for 10 passages) and passage 0 (P0, which was not cultured in hydrogel). These results showed no apparent differences of the mRNA level (Fig. S6).

As 2D cultures are widely used for drug discovery, we tested whether TICs expanded in 3D hydrogel could be returned to standard 2D surfaces. After 10 passages in 3D hydrogels, dissociated TICs attached to surfaces coated with laminin and grew well (Fig. S7). These cells could subsequently undergo long term expansion in 2D, indicating that cells could be interchanged between 2D and this 3D systems as needed.

**Figure 4:**
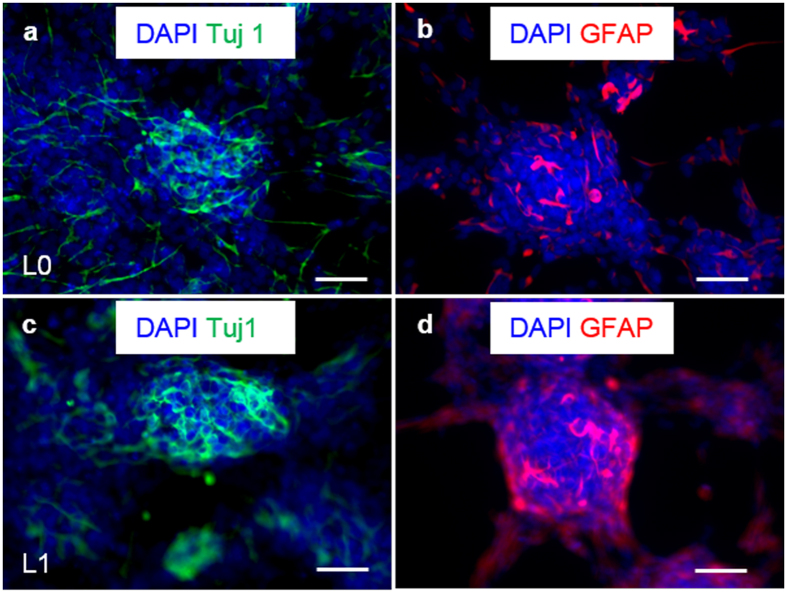
*In vitro* differentiation of glioblastoma TICs. After 10 passages in the 3D thermorevesible PNIPAAm-PEG hydrogel, L0 (**a**,**b**) and L1 (**c**,**d**) spheroids were differentiated for 14 days and stained with antibodies against neuron marker, Tuj1 and glia cell marker, GFAP. Scale bar: 50 μm.

**Figure 5:**
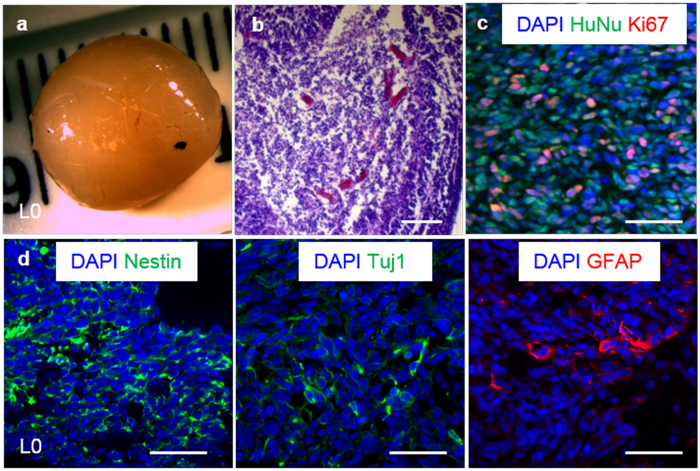
Xenotransplantation of glioblastoma TICs. After 10 passages in the 3D thermorevesible PNIPAAm-PEG hydrogel, L0 TICs were transplanted subcutaneously to the NOD-SCID mice. (**a**) Harvested tumor. (**b**) H&E staining of the tumor section. (**c**) Majority of the cells in the tumor tissue were human nuclear antigen (HuNu) positive human cells and large percentage of cells were proliferating (Ki67+). (**d**) Nestin+ TICs, Tuj1+ neurons and GFAP+ glia cells were found in the tumor tissue. Scale bar: 50 μm.

To further establish maintenance of the stem cell properties during long-term expansion in the 3D thermoreversible hydrogel system, TICs were plated on 2D surfaces and differentiated after 10 passages within the hydrogel. Both L0 and L1 could be differentiated into Tuj1+ neural cells and GFAP+ glial cells (Fig. 4). After long-term culturing in the 3D hydrogel, both L0 and L1 TICs initiated tumors after xenotransplantation into the immunodeficient NOD-SCID mice. Histological analysis showed that the majority of cells within the tumor were HuNu+ (human nuclear antigen) human cells, and that a large percentage was Ki67+ proliferating cells. Nestin+ TICs, differentiated Tuj1+ neurons and GFAP+ glial cells were detected in the tumor (Figs 5 and S8).

**Figure 6:**
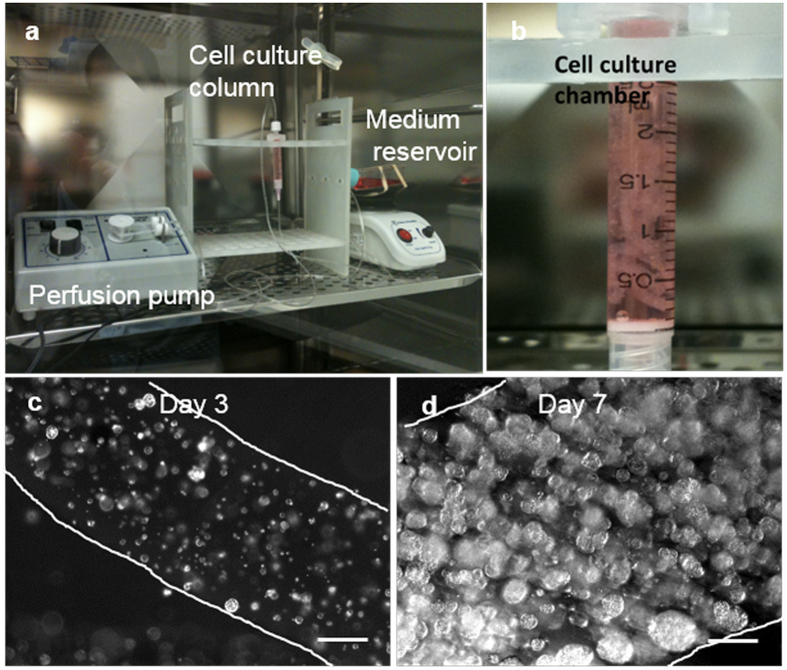
A scalable bioreactor for producing glioblastoma TICs. TICs were mixed with PNIPAAm-PEG solution and processed into fibers. The fibers were suspended in a chromatography column. Cell culture medium was continuously perfused through the column. (**a**) A picture showing the column, pump and medium reservoir of the bioreactor. (**b**) A close look of the column with hydrogel fibers. Phase pictures showing one hydrogel fiber on day 3 (**c**) and day 7 (**d**) of the culture. The edges of the fibers were outlined with white lines. The individual spheroids were clearly seen in the fibers. Scale bar: 250 μm.

We further developed a prototype bioreactor to demonstrate the application of thermoreversible hydrogels for the scalable manufacturing of glioblastoma TICs (Fig. 6). Single TICs were mixed with 10% PNIPAAM-PEG solution and processed into microfibers with an extruder. Fibers with cells were suspended within a column statically. Medium was perfused through the bioreactor with a pump. Single cells grew into spheroids. 5 × 10^7^ TICs were readily generated within 2.5 ml hydrogel in the bioreactor (Fig. 6).

## Discussion

In this study, we successfully cultured multiple primary glioblastoma TICs in thermoreversible PNIPAAM-PEG hydrogels, achieving high growth rate (~20-fold expansion/7days) and high volumetric yield (~2.0 × 10^7^ cells/ml) (Figs 1, 2 and 3). We demonstrated that TICs could be cultured in the PNIPAAM-PEG hydrogel for long term (~50 days, 10 passages with accumulative ~>10^10^-fold expansion tested in our laboratory), and they retained the expression of stem cell marker (>95% Nestin+) and the capability to differentiate into neurons and glia cells (Fig. 4). TICs initiated new tumors in the NOD-SCID mice following xenotransplantation (Figs 5 and S8). TICs cultured in the 3D PNIPAAM-PEG gel for long term were easily returned to conventional 2D culture systems (Fig. S7). In addition, the PNIPAAM-PEG gel culture system was both scalable and easily automated.

Large numbers of TICs are required for drug screening and therapeutic development^11^,^13^,^20^,^21^,^22^,^23^,^24^. In a typical drug screening with 384-well plates, ~800 cells are plated in each well. Typically, four concentrations for each compound and triplicates for each sample are tested, requiring around 10^10^ TICs to screen a library with one million compounds once. At a typical culture density, around 12 × 10^6^ TICs can be harvested from one 6-well plate. Around 833 6-well plates are needed to generate 1 × 10^10^ TICs. Maintaining 833 plates clearly requires large incubator space, labor, time, cell culture reagents, and cost. In addition, the plate-to-plate and batch-to-batch variations are large in 2D culture systems. For these reasons, 2D culture systems are not suitable for large scale cell manufacturing.

3D dynamic suspension culture systems, such as the stirred-tank bioreactor or spinner flasks, are widely used for large-scale mammalian cell culture in the biopharmaceutical industry. Our results demonstrated that a maximum volumetric yield around 1× to 2 × 10^6^ cells could be achieved with these systems (Fig. S3). It would require a 5 liter to 10 liter bioreactor to produce 1 × 10^10^ TICs^16^. In addition, significant cell death was common in dynamic suspension culture systems (Fig. S3), leading to low quality of the produced cells. To date, no studies have shown that TICs could be cultured long term in these dynamic suspension culture systems.

Our previous research on culturing human pluripotent stem cells and other stem cells showed the shear force generated from the medium flow induced significant cell death^18^. Theoretically, shear force can be avoided by culturing cells statically. The problem is that cells can easily agglomerate without shearing. The supply of oxygen, nutrients and growth factors to cells located at the center of cell agglomerates larger than 500 μm in diameter becomes limited, leading to their slow growth, death and uncontrolled differentiation^25^,^26^,^27^,^28^. Culturing cells in the 3D PNIPAAM-PEG hydrogels eliminates these limitations. Hydrogel scaffolds provide sufficient space for cell growth and act as physical barriers to prevent cell agglomeration and shear force, leading to high cell growth rate at high density, as well as high cell quality^18^,^19^ (Figs 1, 2 and 3). Our data showed above 95% of cells in the hydrogel were viable and single cells grew into uniform spheroids (Figs 1 and 3). The cell growth kinetics, the expressions of glioblastoma TICs marker Nestin, the differentiation markers, GFAP and Tuj1, and the proliferation marker, Ki67 were very similar between the passage 1 and 10 glioblastoma TICs (Figs 1 and 3). Additionally, we analyzed the mRNA level of CD133, CD44, CD15 and CD49f, which have been used as markers for glioblastoma TICs^5^. Results indicated that there is no significant difference of CD133, CD44, CD15 and CD49f mRNA expression between passage 10 and passage 0 (Fig. S6). These data indicated that the hydrogel did not significantly change the phenotypes of the cells, neither selected subclones.

Our results demonstrated that single TICs grew in the PNIPAAM-PEG gel through clonal expansion (Fig. 2). The neighboring TICs spheroids were isolated by the hydrogel scaffold and rarely fused or merged. While in the neurosphere culture or the dynamic suspension culture, single TICs first clustered into small aggregates and then expanded. These aggregates could fuse or merge along the whole culture. Thus, the PNIPAAM-PEG hydrogel culture system provides a valuable tool for clonally studying TICs^29^,^30^,^31^.

We built a prototype bioreactor to demonstrate that the PNIPAAM-PEG hydrogels are compatible with automation (Fig. 6). Automated production significantly reduces manufacturing cost and batch-to-batch variance. In summary, the PNIPAAM-PEG hydrogel culture system offers several advancements over the current state-of-art for culturing glioblastoma TICs. We expect this culture system will expand the use of TICs for drug development.

## Methods

### Materials

Cell culture reagents and their supplies: laminin (Invitrogen); Heparin (Sigma); EGF (R&D); FGF (R&D); DNAse (Roche); Trypsin inhibitor (SIGMA); Accutase (Invitrogen); Trypsin-EDTA 0.05% (Invitrogen); Neurocult^TM^ NS-A Proliferation kit (Stem Cell Technologies); MEM medium (Gibco); Matrigel (BD Biosciences); PNIPAAm-PEG (Mebiol^®^ Gel, Cosmo Bio, USA); LIVE/DEAD^®^ Cell Viability staining (Invitrogen). Tuj1 (1:10,000; Sigma); Nestin (1:200; Millipore); Ki-67 (1:500; Invitrogen); anti-glial fibrillary acidic protein (1:500; Dako). TRIzol (Ambion); Maxima first strand cDNA synthesis Kit (Thermo Fisher Scientific); Power SYBR Green Master Mix (Applied biosystems); all primers were synthesized from Integrated DNA technologies.

### Primary Cells

All experiments were performed in accordance with the national regulations. All experiments were approved by the Institutional Review Board (IRB) at University of Florida. All patients gave their informed consent prior to the experiments. Fresh brain tumor samples were obtained at the time of surgical excision. Brain tumors were dissociated into single cells with trypsin and cultured in suspension as neurospheres in Neurocult^TM^ medium to establish the cell lines^6^.

### 2D Adherent Culture

Confluent cells were treated with trypsin at 37 °C in the incubator for 2–3 min and dissociated into single cells. Equal volume of trypsin inhibitor were added to inactivate the trypsin. 1 × 10^5^ cells in 2 ml of Neurocult^TM^ medium supplemented with 10 ng/ml bFGF, 20 ng/ml EGF and 2 μg/ml heparin was plated in a well of the 6-well plate that were pre-coated with 10 μg/ml laminin at 37 °C in an incubator for minimum of 3 hours^1^.

### 3D Neurosphere Culture

Neurospheres were collected via centrifuging at 100 g for 5 min at room temperature and treated with trypsin at 37 °C for 5 min. Trypsin inhibitors were then added to inactivate the trypsin. Cells were dissociated into single cells with pipettes. 2.5 × 10^5^ cells in 5 ml of Neurocult^TM^ medium supplemented with 10 ng/ml bFGF, 20 ng/ml EGF and 2 μg/ml heparin were replated in a non-treated T-25 Flask. To change medium, the flask was tilted and placed in tissue culture hood for 5 minutes to let the neuronspheres sediment. 90% of the old medium was removed and replaced with fresh medium. When the diameters of neurosphere approached ~150 μm, neurospheres were ready for the next passaging^5^.

### 3D Culture in Shaking Plates

Single TICs were suspended in Neurocult^TM^ medium supplemented with 10 ng/ml bFGF, 20 ng/ml EGF and 2 μg/ml heparin in low adhesion 6-well plate. The plate was shaken at 75–90 rpm. Medium was changed daily. To change medium, the plate was tilted and placed in tissue culture hood for 5 minutes to let the neuronspheres sediment. 90% of the old medium was removed and replaced with fresh medium^18^.

### Culturing Glioblastoma TICs in 3D Thermoreversible Hydrogels

Single glioblastoma TICs were mixed with 10% PNIPAAm-PEG solution at 4 °C and cast on tissue culture plate, then incubated at 37 °C for 15 mins to form hydrogels before adding warm Neurocult^TM^ medium supplemented with 10 ng/ml bFGF, 20 ng/ml EGF and 2 μg/ml heparin. Medium was changed daily and cells were passaged every 5 days. To passage cells, ice cold PBS was added to the 3D culture for 2 minutes to dissolve the hydrogel. Spheroids were collected by centrifuging at 200 g for 3 minutes and treated with trypsin at 37 °C for 5 min. Trypsin inhibitors were then added to inactivate the trypsin. Cells were dissociated into single cells with pipettes. The NucleoCounter NC-200 (Chemometec) was used to count cell numbers. The dissociated TICs could be encapsulated into the hydrogel for the next round of growth^19^.

### Quantitative real time polymerase chain reaction (qRT-PCR)

Total RNA was extracted with TRIzol reagent. cDNA was synthesized using Maxima first strand cDNA synthesis Kit according to the manufacturer’s instructions. qRT-PCR was performed using Power SYBR Green Master Mix. Amplification was conducted as follows: 95 °C for 5 min, 40 cycles at 95 °C for 30 s, 60 °C for 20 s and 72 °C 30 s. Experiments were performed in triplicate. The 2^−∆∆Ct^ method was employed to calculate the specific gene fold change of P10/P0, where ∆∆Ct = (Ct _target P10_ − Ct _GAPDH P10_) − (Ct _target P0_ − Ct _GAPDH P0_). All primers for CD133, CD44, CD15, CD49f and GAPDH were summarized in Table S1.

### Culturing Glioblastoma TICs in the Column Bioreactor

To prepare hydrogel fibers, a 4 °C PNIPAAm-PEG solution containing cells was extruded through an around 1 mm diameter tube into room temperature Neurocult^TM^ medium in a chromatography column. The resulting hydrogel fibers were cultured in suspension in Neurocult^TM^ medium supplemented with 10 ng/ml bFGF, 20 ng/ml EGF and 2 μg/ml heparin at 37 °C. Medium was continuously perfused into the column.

### *In vitro* differentiation

For 2D differentiation, 3D TICs spheroids were plated onto laminin-coated plates and cultured in Neurocult^TM^ medium without bFGFs, EGFs and heparin for 14 days. For in gel 3D differentiation, TICs spheroids were cultured in Neurocult^TM^ medium without bFGFs, EGFs and heparin for 14 days.

### Xenotransplantation

All animal protocols were approved by the Animal Care and Use Committee of the University of Nebraska, Lincoln. All experimental procedures involving animals were carried out in accordance with the guidelines of the Institutional Animal Care and Use Committee of the University of Nebraska, Lincoln. 2 × 10^6^ glioblastoma TICs were suspended in 25 μl PBS + 25 μl Matrigel and injected subcutaneously at the back of the neck of the NOD-SCID mice (Charles River Laboratory), and tumors were harvested when sizes reached 1.0 cm. The tumor was then fixed with 4% PFA for 48 hours and cut into two halves. One half was dehydrated with 70%, 95%, and 100% ethanol sequentially, and de-fated with xylene for 2 hours before embedding in paraffin. 10 μm thick sections were cut and stained with hematoxylin and eosin. The second half was soaked in 20% sucrose for one week, embedded in OCT compounds and frozen. The tumor tissue was then cryosectioned for immunostaining.

### Immunostaining and imaging

Cells cultured on 2D surfaces were fixed with 4% paraformaldehyde (PFA) at room temperature for 15 minutes, permeabilized with 0.25% Triton X-100 for 10 min, and blocked with 5% goat serum for 1 hour before incubating with primary antibodies at room temperature for 2 hours. After extensive washing, secondary antibodies in 2% BSA were added and incubated for another 1 hour. Cells were washed with PBS for 3 times before imaging.

To stain 3D spheroids, spheroids were harvested and fixed with 4% PFA at room temperature for 30 minutes, then incubated with PBS + 0.25% Triton X-100 + 5% goat serum + primary antibodies at 4 °C for 48 hours. After extensive washing, secondary antibodies in 2% BSA was added and incubated at 4 °C for 4 hours. Cells were washed with PBS for 3 times before imaging. Spheroids were imaged with Nikon A1 confocal system. Images were analyzed with Image J.

LIVE/DEAD^®^ Cell Viability staining was used to assess live and dead cells, according to the product manual.

### Statistical analysis

Statistical analyses were done using the statistical package Instat (GraphPad Software, La Jolla, CA). For multiple comparisons, the means of triplicate samples were compared using the Tukey multiple comparisons analysis with the alpha level indicated in the figure legend.

## Additional Information

**How to cite this article**: Li, Q. *et al*. Scalable Production of Glioblastoma Tumor-initiating cells in 3 Dimension Thermoreversible Hydrogels. *Sci. Rep.* **6**, 31915; doi: 10.1038/srep31915 (2016).

## Supplementary Material

Supplementary Information
