# Supplementary material for: Scalable Production of Glioblastoma Tumor-initiating Cells in 3 Dimension Thermoreversible Hydrogels
Source: Sci Rep. 2016 Aug 23;6:31915. doi: 10.1038/srep31915 (PMC4994035; doi:10.1038/srep31915)
Supplement: Supplementary Information [file srep31915-s1.pdf]

Scalable Production of Glioblastoma Tumor-initiating Cells in 3 Dimension Thermoreversible Hydrogels

Qiang Li[1,a], Haishuang Lin[1,a], Ou Wang[1], Xuefeng Qiu[2], Srivatsan Kidambi[1,3,4,5,6], Loic P. Deleyrolle[7], Brent A. Reynolds[7] and Yuguo Lei[1,3,4,*]

1: Department of Chemical and Biomolecular Engineering, University of Nebraska, Lincoln, Nebraska, USA
2: Department of Bioengineering, University of California, Los Angeles, Los Angeles, California, USA
3: Mary and Dick Holland Regenerative Medicine Program, University of Nebraska Medical Center, Omaha, Nebraska, USA
4: Fred & Pamela Buffett Cancer Center, University of Nebraska Medical Center, Omaha, Nebraska, USA
5: Nebraska Center for Materials and Nanoscience, University of Nebraska, Lincoln, Nebraska, USA
6: Nebraska Center for the Prevention of Obesity Diseases, University of Nebraska, Lincoln, Nebraska, USA
7: Department of Neurosurgery, University of Florida College of Medicine, McKnight Brain Institute, Gainesville, Florida, USA

a: These authors contributed equally

* Corresponding Author
Yuguo Lei
820 N 16th St
Lincoln, NE 68588
Email : ylei14@unl.edu
Phone: 402-472-5313
Fax: 402-472-6989

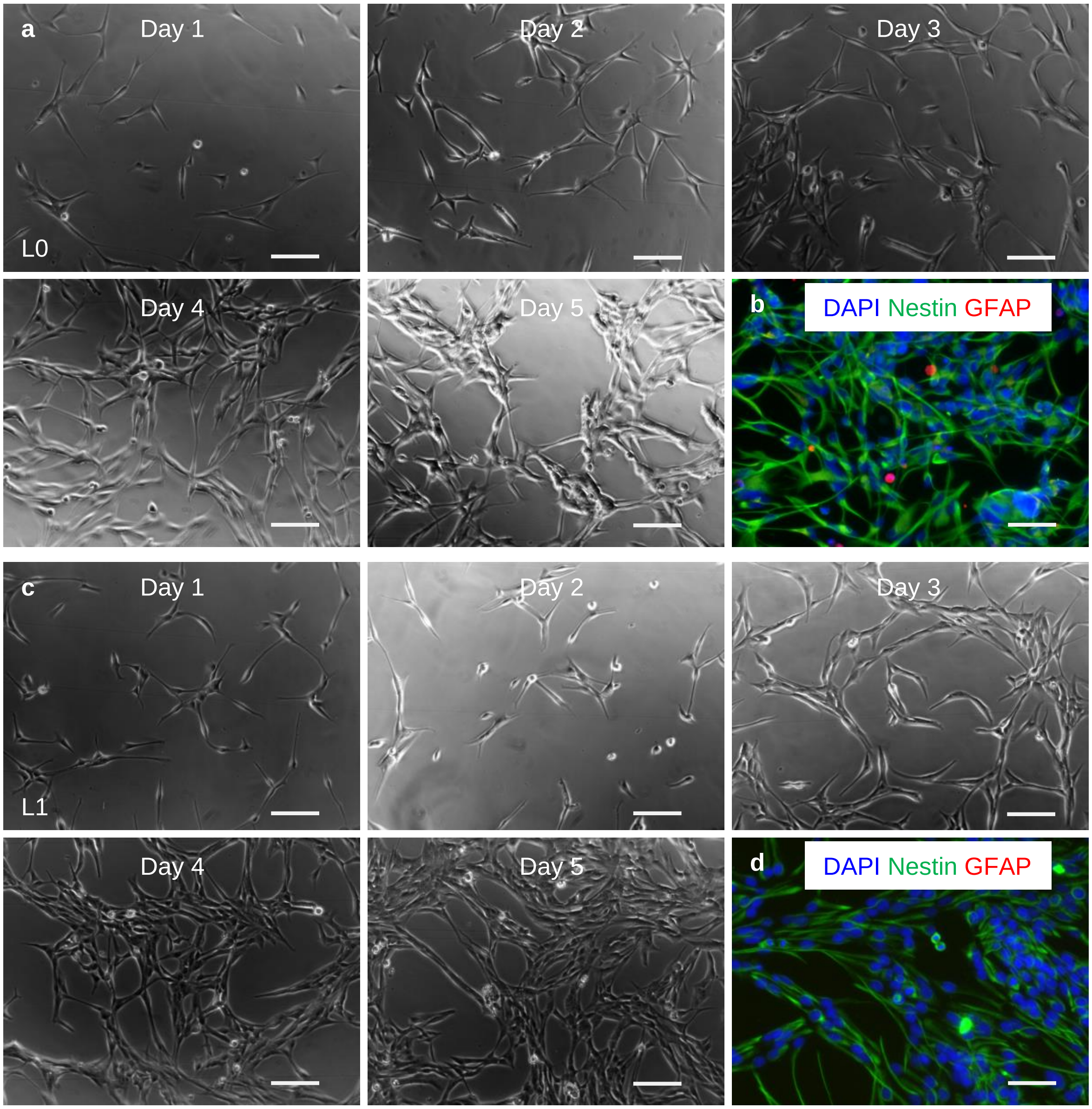

Figure S1. Culturing glioblastoma TICs as monolayer in 2D. L0 (**a**) and L1 (**c**) TICs were cultured on laminin-coated 6-well plates for 5 days. (**b, d**) Majority of the cells were Nestin+ stem cells. Very few or no differentiated GFAP+ cells were detected. Scale bar: (**a, c**) 100 µm; (**b, d**) 50 µm.

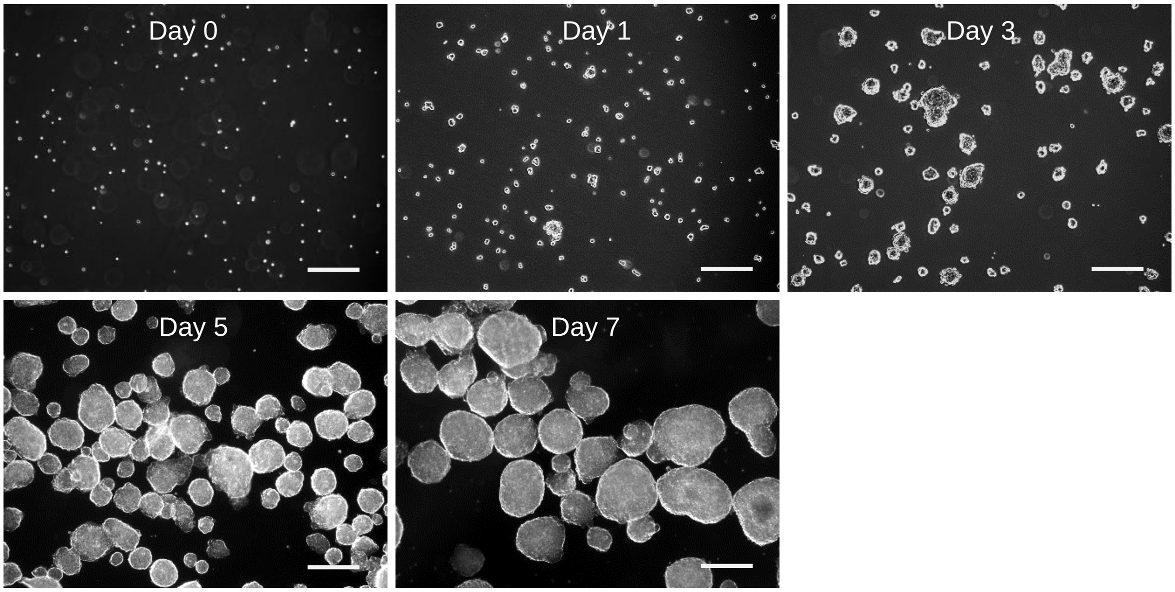

Figure S2. Culturing glioblastoma TICs as neurospheres. L0 TICs were suspended in liquid medium statically at $5x10^4$ cells/ml. Phase pictures of the growing spheroids were shown. Scale bar: 250 µm.

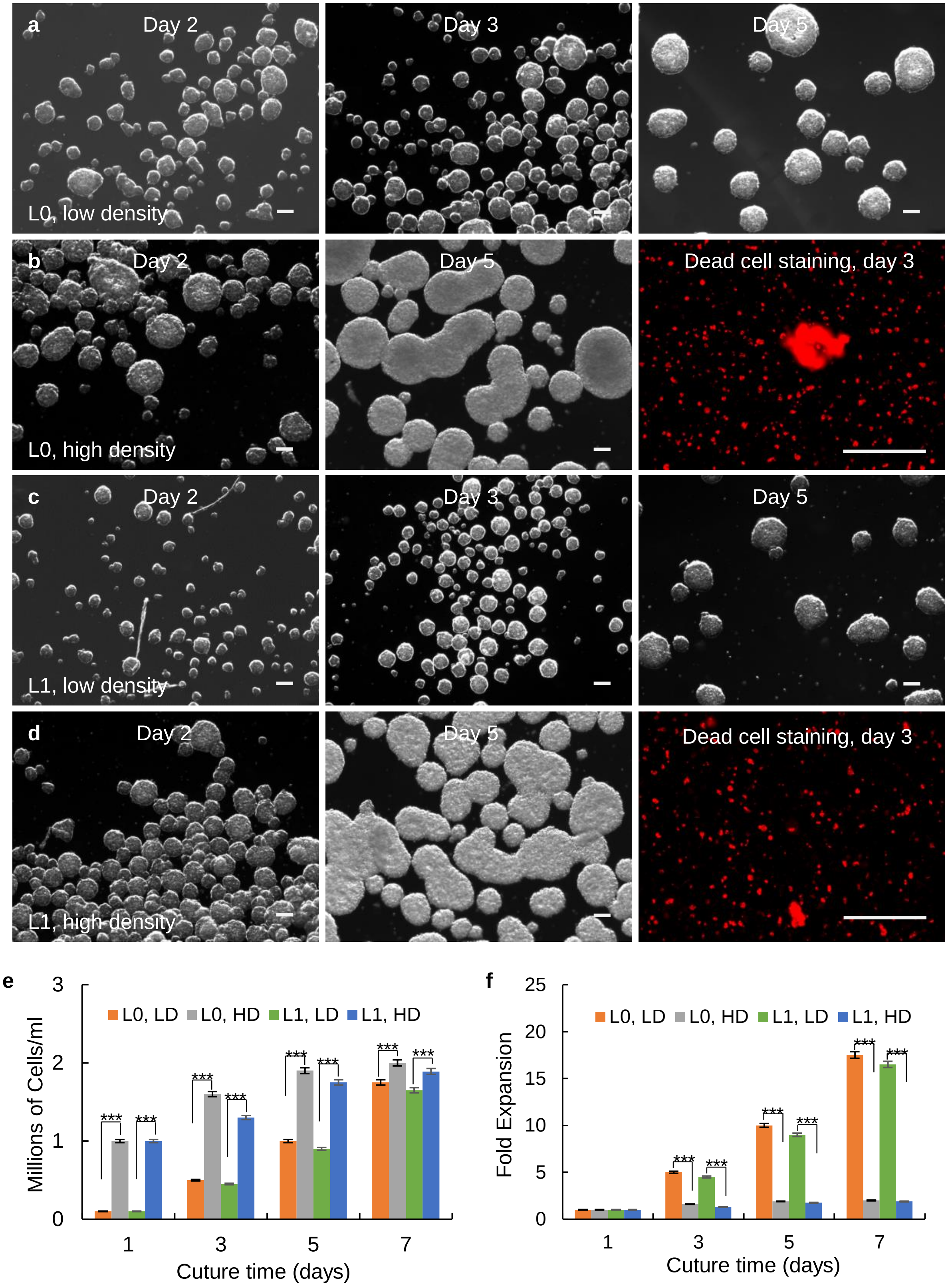

Figure S3. Culturing glioblastoma TICs in shaking plates. L0 and L1 TICs were suspended in liquid medium at $1x10^5$ (low density, LD) and $1x10^6$ (high density, HD) cells/ml in low adhesion plates on a orbital shaker at 75-90 rpm. Phase pictures and dead cell staining of TICs were shown (**a, b, c, d**). The cell density and fold of expansion were quantified (**e, f**). Error bars represent the standard deviation (n=3). *** indicates statistical significance at a level of p<0.001. Scale bar: 100 µm.

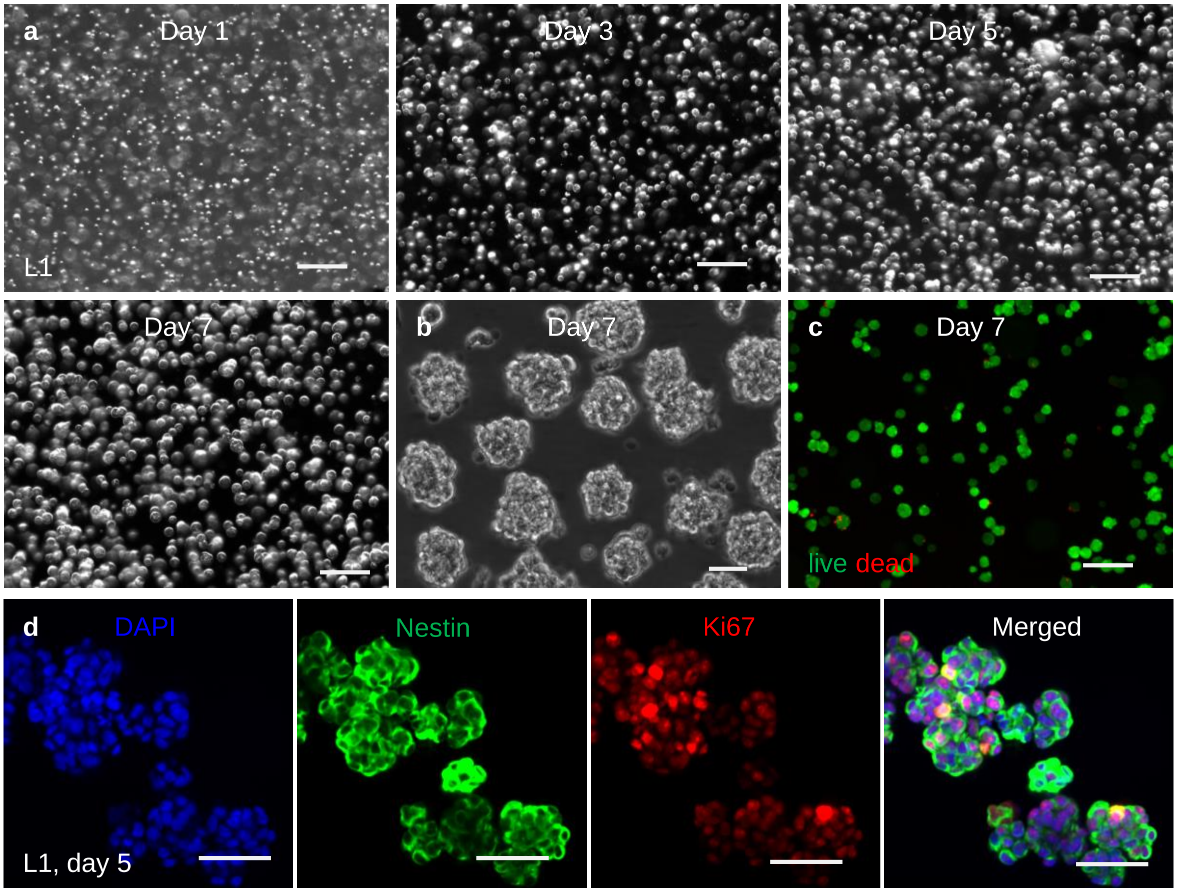

Figure S4. Culture glioblastoma TICs in 3D thermorevesible PNIPAAm-PEG hydrogel. (**a**) phase images showing L1 spheroids in the hydrogel on day 1, 3, 5, 7 of the culture. (**b**) Day 7 L1 spheroids released from the hydrogel. (**c**) Live (green) and dead (red) staining of day 7 L1 spheroids. (**d**) Immunostaining of day 5 L0 spheroids. Images were taken after spheroids were released from the hydrogel in (**b, c**). Scale bar: (**a**, **c**) 250 µm, (**b**, **d**) 50 µm.

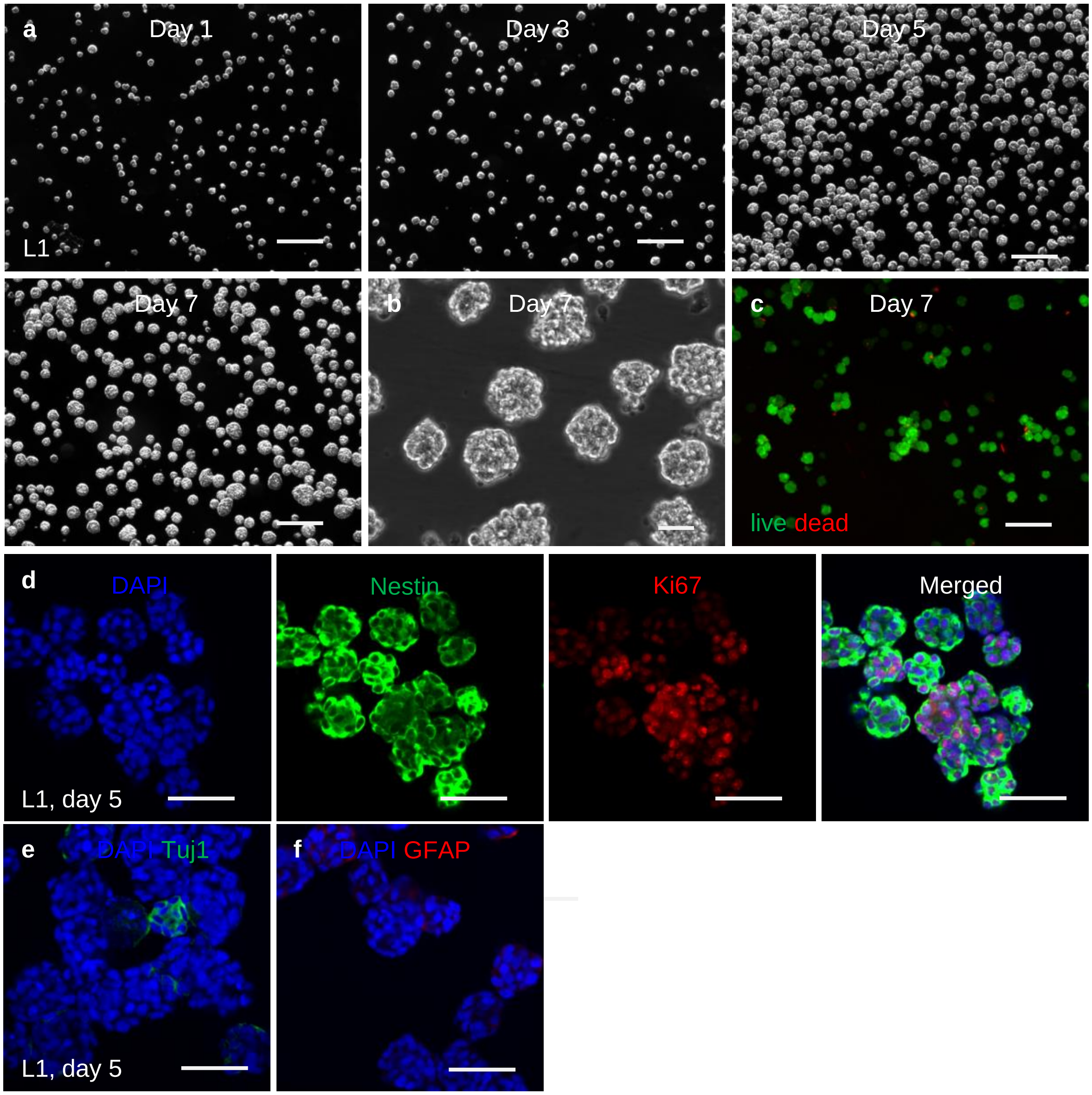

Figure S5. Long-term culture of glioblastoma TICs in 3D thermorevesible PNIPAAm-PEG hydrogel. (**a**) phase images showing L1 spheroids in the hydrogel on day 1, 3, 5, 7 of the culture at passage 10. (**b**) Day 7 L1 spheroids released from the hydrogel at passage 10. (**c**) Live (green) and dead (red) staining of day 7 L1 spheroids at passage 10. (**d, e, f**) Immunostaining of day 5 L0 spheroids at passage 10. Images were taken after the spheroids were released from the hydrogels. Scale bar: (**a**, **c**) 250 µm, (**b**, **d**, **e**, **f**) 50 µm.

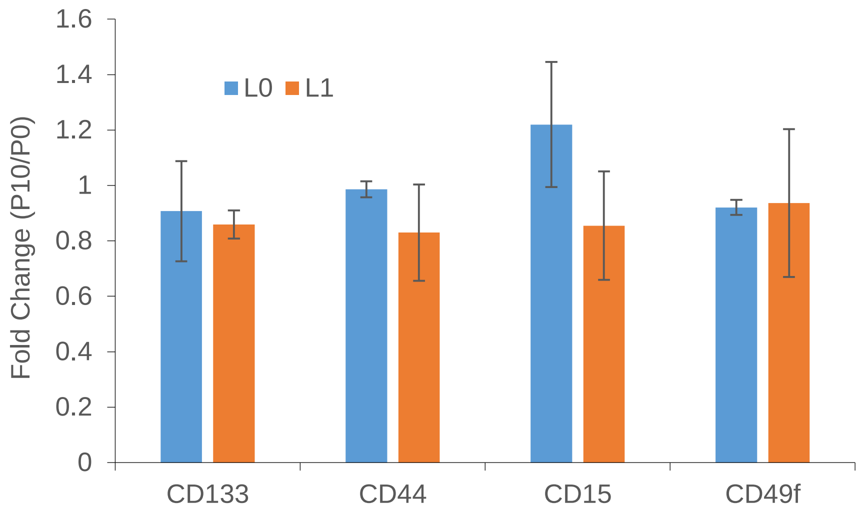

Figure S6. qRT-PCR on the mRNA level of CD133, CD44, CD15, CD49f. The ratio of their expression at passage 10 and passage 0 were shown. Error bars represent the standard deviation (n=3).

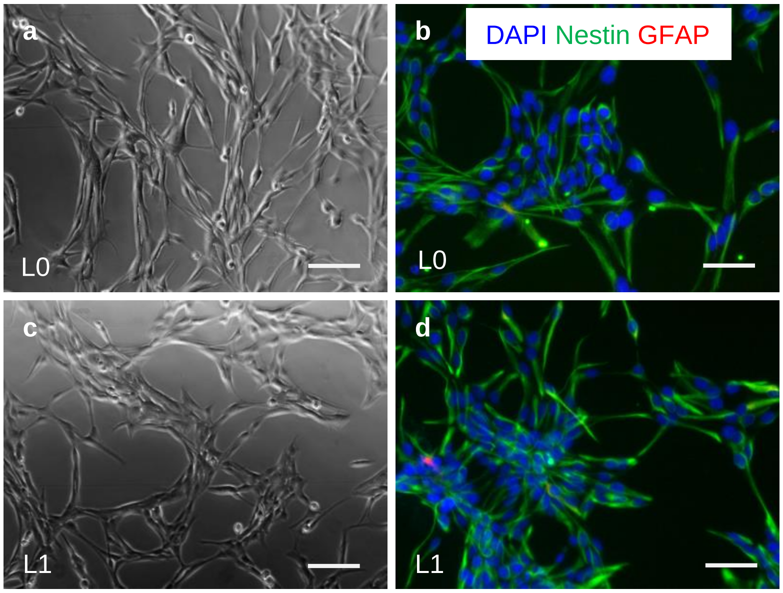

Figure S7. Returning to the 2D culture. After 10 passages in the 3D thermorevesible PNIPAAm-PEG hydrogel, L0 (**a, b**) and L1 (**c, d**) TICs were plated to the laminin-coated 2D surface. Cells on day 5 were shown. Scale bar: (**a**, **c**) 250 µm, (**b**, **d**) 50 µm.

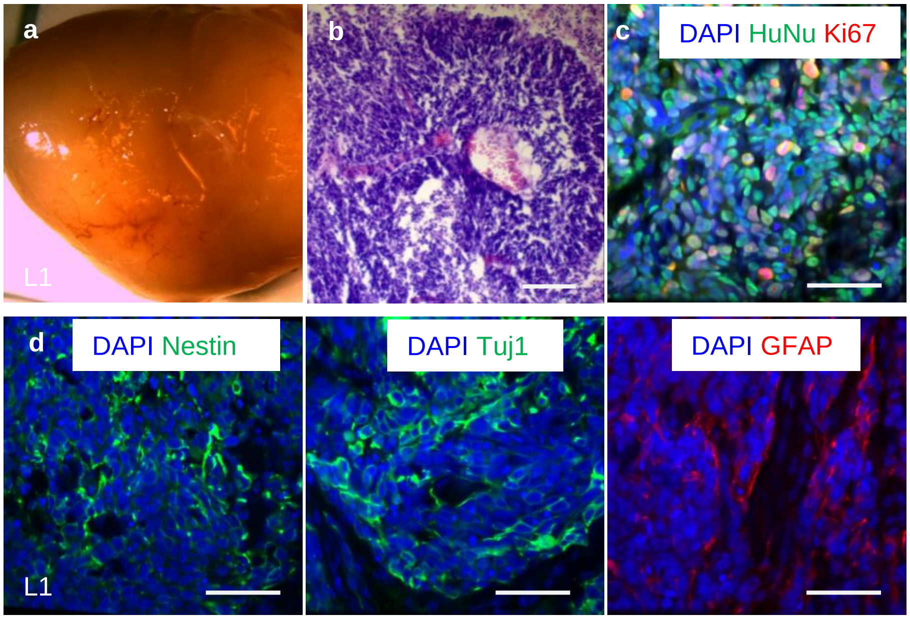

Figure S8. Xenotransplantation of glioblastoma TICs. After 10 passages in the 3D thermorevesible PNIPAAm-PEG hydrogel, L1 TICs were transplanted subcutaneously to the NOD-SCID mice. (**a**) Harvested tumor. (**b**) H&E staining of the tumor section. (**c**) Majority of the cells in the tumor tissue were human nuclear antigen (HuNu) positive human cells and large percentage of cells were proliferating (Ki67+). (**d**) Nestin+ TICs, Tuj1+ neurons and GFAP+ glia cells were found in the tumor tissue. Scale bar: 50 µm.

Table S1. Primer Sequences for qRT-PCR.

Gene Name	Sequences (5'-3')	References
CD133	F: GCGTGATTTCCCAGAAGATA	1
	R: CCCCAGGACACAGCATAGAA	
CD44	F: CAACTCCATCTGTGCAGCAAA	2
	R: GTAACCTCCTGAAGTGCTGCTC	
CD15	F: CTTTGTGCCTTATGGCTACC	3
	R: TTGGCTCAGTTGGTGGTAGT	
CD49F	F: CGAGTGACTGTGTTTCCCTCA	4
	R: GCATCAAGATCCCAGCGAGA	
GAPDH	F: TCGACAGTCAGCCGCATCTTCTTT	5
	R: ACCAAATCCGTTGACTCCGACCTT	

References

1. Ricci-Vitiani, L. *et al.* Identification and expansion of human colon-cancer-initiating cells. *Nature* **445,** 111–115 (2007).

2. Wei, K. *et al.* Evaluation of the prognostic value of CD44 in glioblastoma multiforme. **260,** 253–260 (2010).

3. Dewi, Y., Shahib, M., Boesoirie, T. & Achmad, D. Studied of CD14 and CD15 gene expressions for myeloid derived suppressor cell profile at the RNA level as a predictor for progressivity in nasopharyngeal carcinoma. **4,** 6 (2016).

4. Ka, L., Kong, H., Cell, S., Shing, L. K. & Surgery, O. CD49f acts as an inflammation sensor to regulate differentiation, adhesion, and migration of human mesenchymal stem cells. *Stem Cells* **33,** 2798–2810 (2015).

5. Liu, Y. *et al.* Medial ganglionic eminence-like cells derived from human embryonic stem cells correct learning and memory deficits. *Nat. Biotechnol.* **31,** 440–447 (2013).
